# Adverse events profile associated with intermittent fasting in adults with overweight or obesity: a systematic review and meta-analysis of randomized controlled trials

**DOI:** 10.1186/s12937-024-00975-9

**Published:** 2024-07-10

**Authors:** Fan Zhong, Ting Zhu, Xingyi Jin, Xiangjun Chen, Ruipeng Wu, Li Shao, Shaokang Wang

**Affiliations:** 1 Ulink College of Shanghai, Shanghai, 201615 China; 2 Key Laboratory of Environmental Medicine and Engineering of Ministry of Education, and Department of Nutrition and Food Hygiene, School of Public Health, Southeast University, Nanjing, 210009 China; 3 Department of Clinical Nutrition, Children’s Hospital of Nanjing Medical University, Nanjing, 210008 China; 4 Clinical Medical Research Center for Plateau Gastroenterological disease of Xizang Autonomous Region, and School of Medicine , Xizang Minzu University, Xianyang, 712082 China

**Keywords:** Adverse events, Intermittent fasting, Overweight, Obesity, Meta-analysis, Randomized controlled trials

## Abstract

**Background:**

There is little evidence to comprehensively summarize the adverse events (AEs) profile of intermittent fasting (IF) despite its widespread use in patients with overweight or obesity.

**Methods:**

We searched the main electronic databases and registry websites to identify eligible randomized controlled trials (RCTs) comparing IF versus control groups. A direct meta-analysis using a fixed-effect model was conducted to pool the risk differences regarding common AEs and dropouts. Study quality was assessed by using the Jadad scale. Pre-specified subgroup and sensitivity analyses were conducted to explore potential heterogeneity.

**Results:**

A total of 15 RCTs involving 1,365 adult individuals were included. Findings did not show a significant difference between IF and Control in risk rate of fatigue [0%, 95% confidence interval (CI), -1% to 2%; *P* = 0.61], headache [0%, 95%CI: -1% to 2%; *P* = 0.86] and dropout [1%, 95%CI: -2% to 4%; *P* = 0.51]. However, a numerically higher risk of dizziness was noted among the IF alone subgroup with non-early time restricted eating [3%, 95%CI: -0% to 6%; *P* = 0.08].

**Conclusions:**

This meta-analysis suggested that IF was not associated with a greater risk of AEs in adult patients affected by overweight or obesity. Additional large-scale RCTs stratified by key confounders and designed to evaluate the long-term effects of various IF regimens are needed to ascertain these AEs profile.

**Supplementary Information:**

The online version contains supplementary material available at 10.1186/s12937-024-00975-9.

## Introduction

Currently, obesity and overweight are considered as widespread chronic metabolic diseases. In China, an estimated 34.3% of the adult population is overweight with another 16.4% being obese [1]; in the USA, 33.3% of adults with obesity [2] and in Europe, 34.5% are overweight and 15.8% are obese [3]. The rate of overweight and obesity is continuing to rise domestically and globally [4], which is undoubtedly associated with a concomitant rise in medical and economic costs [5].

A wide range of treatments are available for weight loss, including intensive lifestyle interventions, public health programs, pharmacotherapies and surgical bariatric therapies [6], among which intermittent fasting (IF), an eating pattern involving periods of voluntary abstinence from calories for a period of time, alternating with periods of caloric consumption, has gained public popularity as a feasible and easy-to-adapt dietary strategy [7, 8].

Previous meta-analyses have shown that IF can effectively decrease body weight [9], regardless of various regimens [10], namely time-restricted eating (TRE), the 5:2 diet and alternate day fasting (ADF). Despite its weight-centric effectiveness, many people are concerned with the adverse effects of IF [11], including long-term uncertain safety implications [12]. In recent years, many randomized controlled trials (RCTs) have been conducted in adults with overweight or obesity to investigate the potential effects of IF. Given that only narrative reviews on the adverse events (AEs) profile of IF [13, 14] were found, we aimed to conduct the first comprehensive meta-analysis to quantitatively assess the AEs profile of IF based on these published RCTs.

## Methods

### Search strategy and selection criteria

This review protocol was pre-registered on the International Prospective Register of Systematic Reviews database (https://www.crd.york.ac.uk/PROSPERO/; registration ID: CRD42023488573). RCTs to investigate AEs profile of IF were eligible for inclusion in our analysis, without any restrictions in terms of language or publication date. We electronically searched PubMed, Embase, Web of Science Core Collection and Cochrane Library databases on November 15, 2023, using the following search terms (“intermittent fasting” or “time- restricted feeding” or “time-restricted eating” or “alternate day fasting” or “5:2 dieting”) AND Randomized Controlled Trial. We also further conducted searches on the *ClinicalTrials.gov* register website. The detailed search strategies used for these studies are included in Supplementary Table 2, Additional File 1.

Eligible RCTs had to meet the following inclusion criteria: (i) published as an original article; (ii) study participants are adults with obesity or overweight; (iii) evaluated the effect of any regimen of IF as one of the study interventions compared with the control group; and (iv) reported any data on any AEs and dropouts. Only parallel-arm RCTs were eligible for inclusion. When more than one article reported data from a study with the same registration number, the most updated and relevant study was included.

### Data extraction and risk of bias assessment

The following information was extracted from each eligible study in this meta-analysis: (i) first author’s surname and study country; (ii) publication year; (iii) study size; (iv) study population entry criteria; (v) demographics (age, sex and body mass index [BMI]); (vi) IF regimen; (vii) control group regimen; (viii) treatment duration and follow-up duration; (ix) number of subjects with various AEs and dropouts. The reported AEs were further coded using the 23.1-English version of the Medical Dictionary for Regulatory Activities (MedDRA) at preferred terms and system organ class levels.

Key data were extracted using a standardized data-recording form and the risk for bias was assessed according to the Preferred Reporting Items for Systematic Reviews and Meta-Analyses (PRISMA) guidelines (see Supplementary Table 1, Additional File 1) [15]. Three investigators (F.Z, T.Z and X.J) conducted the study search and screening, data extraction, and risk of bias assessment independently by using the revised Cochrane risk of bias tool for randomized trials (ROB2) [16]. Information was checked and adjudicated independently by an additional investigator (W.S.) until agreement was achieved where needed. We also calculated the Jadad score to assess the quality of the included RCTs [17]. The overall quality of evidence for each outcome was also assessed by two independent investigators (F.Z. and W.S.) using the Grading of Recommendations Assessment, Development, and Evaluation (GRADE) methodology [18].

### Outcomes

The primary outcome in the study was the serious adverse events (SAEs). The secondary outcomes included the most occurred specific AEs and dropouts (named dropout, loss to follow-up, loss of contact or withdrawal for various reasons).

### Statistical analysis

We used the statistical software R 4.3.1 (www.r-project.org*)* along with the ‘meta’ package to conduct the direct meta-analysis [19]. Firstly, a subject-based frequency table by treatment was created to depict each specific adverse event as appropriate. Then distinctions of these binary variables were statistically evaluated using risk difference (RD) with a two-sided 95% confidence interval (CI), displayed by forest plots for each of the common AEs. The various IF or Control regimens were merged separately, and for pooling homogeneous study data, a fixed-effect model was established; otherwise, both fixed-effect and random-effects models (restricted maximum-likelihood [REML] estimator used to explore the between-study variance) following inverse variance method were provided. Sensitivity analysis was conducted as appropriate if BMI data were not clearly available in the RCTs. Continuity correction of 0.5 was utilized in studies with zero cell frequencies as appropriate. Two-sided P values of less than 0.05 were considered statistically significant. Between-study heterogeneity was assessed via *I*^*2*^ (less than 25% for low heterogeneity, within 25% and 50% for moderate heterogeneity, within 50% to 75% for substantial heterogeneity and more than 75% for high heterogeneity) [20] and Q test of Cochran (if *P* < 0.10 for heterogeneity) [21]. Both statistical measures evaluate the percent variability across studies due to heterogeneity instead of chance. Subgroup analyses according to prespecified diabetes mellitus status (Yes versus No), IF timing (early TRE [eTRE] versus non-eTRE) and study treatment duration (short-term [< 6 months] versus long-term [6 or 12 months]) were conducted to ascertain the effects of potential confounders on AEs, where eTRE refers to time restricted eating whose eating window starts in early morning (not later than 10:00 AM) [22].

Funnel plots for common AEs and dropouts were performed and the Egger’s regression test [23] was also used to statistically assess publication bias. Upon request the R codes are available from the authors.

## Results

### Search results

**Fig. 1:**
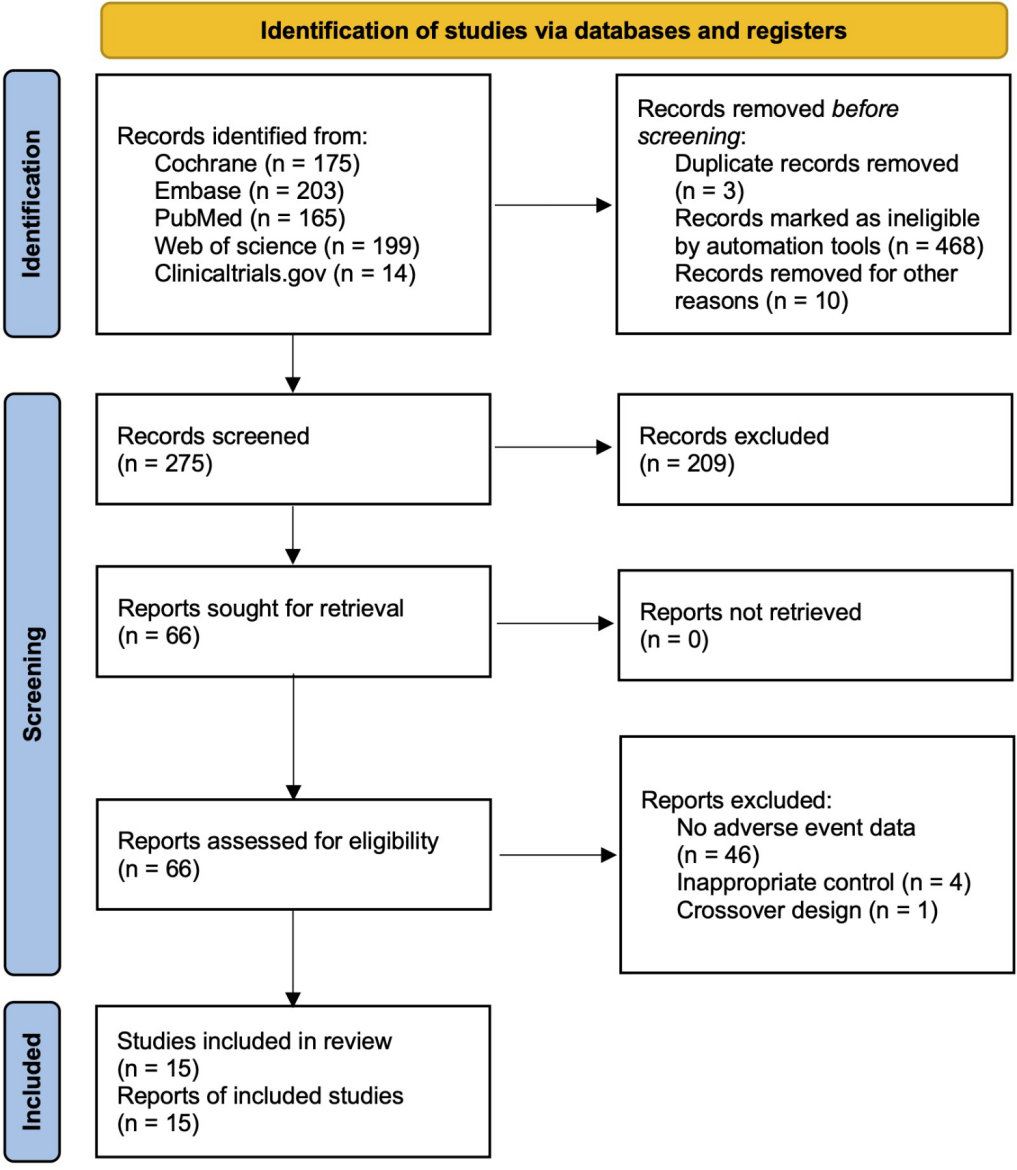
Flow diagram. Flow diagram showing search strategy and inclusion and exclusion of randomized controlled trials for meta-analysis

In total, 756 citations were initially identified with the use of our search strategy, and after duplicate record removal, followed by title and abstract screening, 66 full-text reports were included for final eligibility assessment. Eventually 15 reports originating from 15 RCTs (1,365 individuals) [11, 12, 24–36]met our criteria of inclusion and were involved in this meta-analysis. The whole processes of the relevant study selection are shown in detail in Fig. 1.

### Study characteristics

Table 1 displays the study and participants characteristics for the 15 included trials. Among them, four were conducted in patients with diabetes mellitus [11, 24, 25, 29] and the remaining in patients without diabetes mellitus. Study treatments durations in one-third of the trials were twelve or six months while others ran for no shorter than seven [35] or eight [33, 34] weeks. Various regimens of IF were found, with the fasting to eating ratio ranging from 16:8 h (*n* = 7) or 14:10 h (*n* = 3) daily to 5:2 days (*n* = 3) or 4:3 days (*n* = 2) per week. The mean ages at the study level in included studies were all over thirty years, with the exception of the trial by Liu H et al. (21 ± 1 [mean ± standard deviation] kg/m^2^, but with hidden obesity [body fat percentage ≥ 30%]) [33] and another study with no mean BMI data of subjects reported [25]. Both sexes were included in all trials other than two studies [33, 35]. Hence, the two RCTs [25, 33] were removed from the sensitivity analysis.

**Table 1:** Characteristics of the randomized controlled trials included in the meta-analysis (age and BMI data are presented as mean ± standard deviation.)

First Author (Year) [ No.]	Country	Study size	Study population criteria	Demographics (Age, sex and BMI)	Intermittent fasting regimen (IF alone or only IF in addition to control)	Control group regimen (no any type of IF included)	Treatments/ follow-up Duration
Pavlou, V. (2023) [24]	USA	75	Female or male, aged 18–80 years, and 30 ≤ BMI ≤ 50 kg/m^2^, with type 2 DM	Age: 55 ± 12; Female: 71%; BMI: 39 ± 7	**16:8 TRE**: Ate ad libitum between 12:00 PM and 8:00 PM daily and then fasted from 8:00 PM to 12:00 PM	Reduced energy intake by 25% daily, or maintained usual eating and exercise habits	6/6 months
Obermayer (2023) [25]	Austria	46	Female or male, aged 18–75 years and with insulin-treated type 2 DM	Age: 63 ± 7; Female: 48%; BMI: 34 ± 5	**4:3 diet**: Practiced IF 3 days (Monday, Wednesday, Friday) a week. Reduced calories by 75% and maintained an 18-h period of fasting a day.	Standard care	12 weeks; no further follow-up
Suthutviravut (2023) [26]	Thailand	72	Female or male, aged 18–65 years, BMI ≥ 25 kg/m^2^, with a diagnosis of impaired fasting glucose	Age: 55 ± 8; Female: 70%; BMI: 30 ± 4	**15:9 TRE**: Restricted daily food intake to a 9-h window (8:00 through 17:00), no limit on the types of food and beverages consumed	Usual care	12 weeks; no further follow-up
Chair, S.Y. (2022) [27]	China	101	Female or male, aged 18–65 years, BMI ≥ 23 kg/m^2^, with a diagnosis of prediabetes	Age: 35 ± 6; Female: 63%; BMI: 27 ± 2	**ADF**: Consumed 600 kcal on fasting days and a usual diet on eating days, fasting days alternated with eating days; **16:8 TRE**: Consumed calorie intake during an 8-hour window and then fast for the remaining 16 h each day	Usual care: Received the same individual educational session and maintained usual physical activity	3 weeks/ 3 months
Teong, X.T. (2023) [28]	Australia	209	Female or male, aged 35–75 years, 25 ≤ BMI ≤ 50 kg/m^2^, weight-stable and at increased risk of developing type 2 DM	Age: 58 ± 10; Female: 57%; BMI: 35 ± 5	**4:3 diet**: 30% energy restricted from 8:00 AM to 12:00 PM and followed by 20-h fasting on three non-consecutive days, and ad libitum eating on other five days	CR: 70% of energy requirements daily, without time prescription; or standard care (weight loss booklet provided)	6/12 months
Overland, J. (2018) [29]	Australia	10	Female or male, early middle age and 25 ≤ BMI < 40 kg/m^2^, with type 1 DM	Age: 47 ± 5; Female: 80%; BMI: 32 ± 4	**5:2 diet**: Severe energy restriction on two 24-hour periods per week, with 5 days per week of eating	Continuous moderate energy restriction (30% relative to weight maintenance energy needs)	12 weeks till 12 months
Che, T. (2021) [11]	China	120	Female or male, aged 18–70 years and BMI ≥ 25 kg/m^2^, with DM	Age: 48 ± 9; Female: 46%; BMI: 26 ± 2	**14:10 TRE**: 10-h eating from 8:00 to 18:00 every day	Maintained normal diet	12 weeks; no further follow-up
Liu, D. (2022) [12]	China	139	Female or male, aged 18–75 years, nonsmokers and 28 ≤ BMI < 45 kg/m^2^, no DM	Age: 32 ± 9; Female: 49%; BMI: 32 ± 3	**16:8 TRE**: Eating only between 8:00 and 16:00) with calorie restriction	Calorie-restricted diet of 1500 to 1800 kcal per day for men and 1200 to 1500 kcal for women	12 months; no further follow-up
Sundfør (2018) [30]	Norway	112	Female or male, aged 21–70 years and 30 ≤ BMI < 45 kg/m^2^, no DM	Age: 49 ± 11; Female: 50%; BMI: 35 ± 4	**5:2 diet**: Consumed 400/600 (female/male) on each of two nonconsecutive days a week and then as usual the remaining five days	Reduced energy intake evenly seven days a week	12 months; no further follow-up
Jamshed, H. (2022) [31]	USA	90	Female or male, aged 25–75 years and 30 ≤ BMI ≤ 60 kg/m^2^, no DM	Age: 43 ± 11; Female: 80%; BMI: 40 ± 7	**16:8 TRE**: 8-h eating window from 7:00 AM to 15:00 with energy restriction	Self-selected eating schedule ≥ 12-h window with energy restriction	14 weeks; no further follow-up
Schübel (2018) [32]	Germany	150	Female or male, aged 35–65 years and 25 ≤ BMI < 40 kg/m^2^, nonsmokers, no DM	Age: 50 ± 8; Female: 50%; BMI: 31 ± 4	**5:2 diet**: Restricted energy intake on 2 self-selected non-consecutive days per week to 25% of the individual energy requirement	Consumed ∼80% of the individual energy requirement daily or maintained habitual physical activity levels	12/38 weeks
Liu, H. (2023) [33]	China	77	Female, aged 18–22 years and 18.5 ≤ BMI ≤ 23.9 kg/m^2^, BF% ≥30% (hidden obesity), no DM	Age: 20 ± 2; Female only; BMI: 21 ± 1	**16:8 TRE**: Ate at subjects’ discretion between 10:00 AM and 18:00 daily)	Maintained usual lifestyle or with walking exercise via monitoring	8 weeks; no further follow-up
Kotarsky, C.J. (2021) [34]	USA	23	Female or male, aged 35–60 years and 25.0 ≤ BMI ≤ 34.9 kg/m^2^, no DM	Age: 44 ± 7; Female: 86%; BMI: 30 ± 3	**16:8 TRE**: Consumed all calories between 12:00 PM and 20:00	Maintained dietary habits	8 weeks; no further follow-up
Haganes, K.L. (2022) [35]	Norway	131	Female, aged 18–45 years and BMI ≥ 27 kg/ m^2^, no DM	Age: 36 ± 6; Female only; BMI: 32 ± 4	**14:10 TRE**: ≤10-h TRE daily self-selected window, with ad libitum energy intake	HIIT (three exercise sessions per week), or a non- intervention control group	7 weeks; no further follow-up
Lin, S. (2023) [36]	USA	90	Female or male, aged 18–65 years and 30 ≤ BMI ≤ 50 kg/m^2^, no DM	Age: 44 ± 11; Female: 82%; BMI: 37 ± 5	**16:8 TRE**: 8-h eating between noon and 8:00 PM only, without calorie counting	CR (25% energy restriction daily), or control (eating 10 or more hours per day)	12 months; no further follow-up

*Abbreviations* ADF: Alternate-Day Fasting; BF% : Body Fat Percentage; BMI: Body Mass Index (kg/m^2^); CR: Calorie Restriction; DM: Diabetes Mellitus; HIIT: High-Intensity Interval Training; IF: Intermittent Fasting; TRE: Time-Restricted Eating

### Assessment of bias risk

**Fig. 2:**
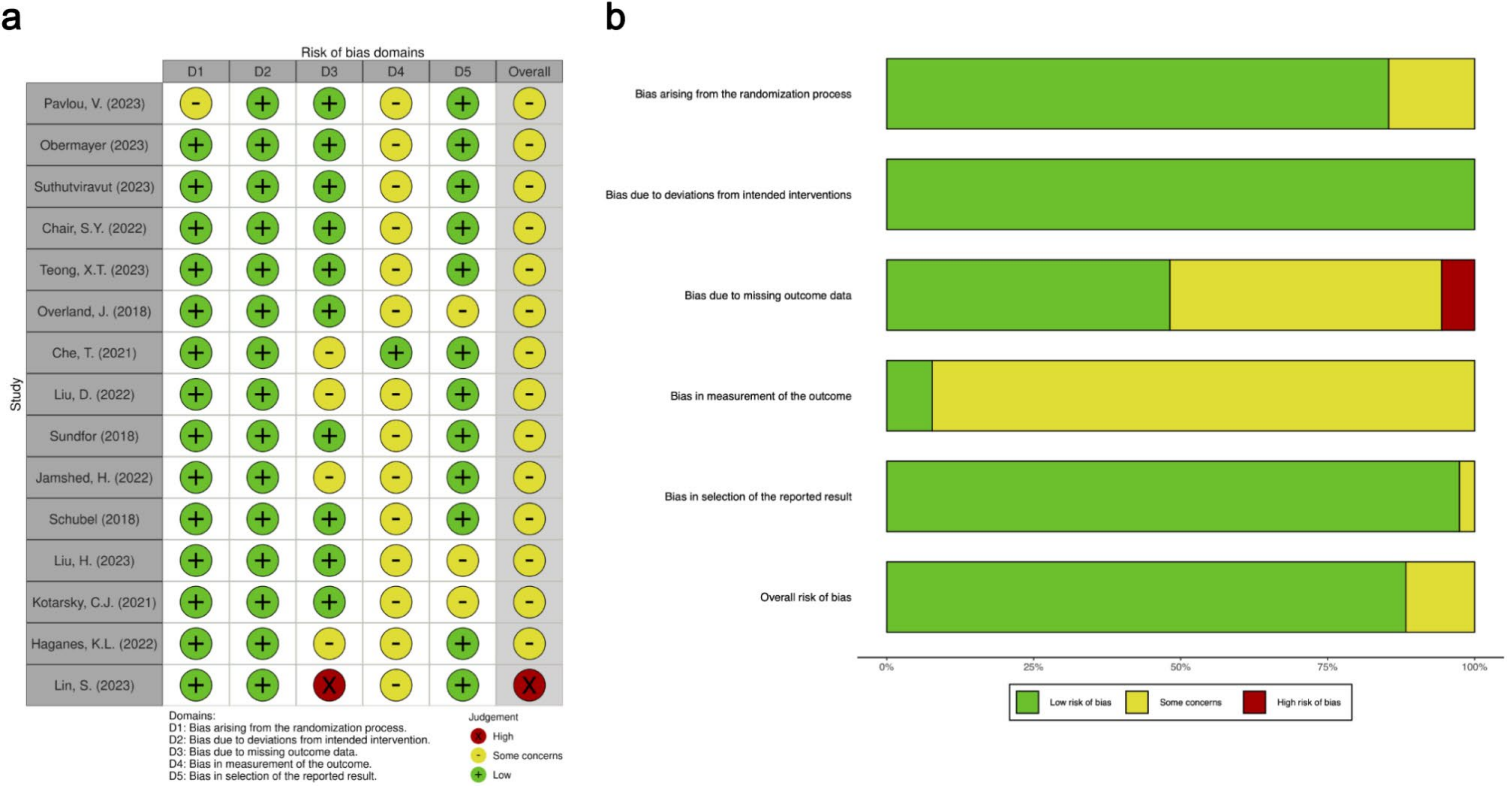
Risk of bias (ROB) assessment in the randomized parallel-arm trials included. (**a**) Traffic light plot of ROB2 assessments; (**b**) Summary plot of ROB2 assessments

Figure 2 presents the assessment for risk of bias in 15 trials according to Cochrane RoB2 tool. 14 out of them had an overall “some concerns” risk of bias, except the “high” risk in Lin, S. et al.’s study [36], and no study stopped early. There were some concerns about the risk of bias in the trial by Kotarsky et al. [34] for lack of mentioning analysis of intention-to-treat principle and the reporting of only TRE-related AEs. Another included RCT [24] did not describe any specific method to generate the randomization sequence. In addition, by using the Jadad score assessment, we noticed that two studies [24, 34] obtained 2 points, one study [11] obtained 4 points and the rest obtained 3 points. In summary, the overall quality of the included RCTs could be largely defined as good.

### Serious adverse events (SAEs)

Six (0.4%) patients in two studies [25, 28] out of the 15 total (1,365 individuals) reported data on any SAEs (2 [0.3%] in the IF group versus 4 [0.5%] in the control group) within the treatment period. All of the reported SAEs were categorized as leading to hospitalization. None of them were considered related to any of the study interventions, and then no specific reported terms were described in the full-text articles. None of the 15 included RCTs reported any major or severe AEs.

### Specific adverse events

Twelve included RCTs (*N* = 1,174) [11, 12, 24, 27–34, 36] reported at least one subject who experienced one of specific AEs, among which the most frequently reported included fatigue, headache, dizziness, constipation and diarrhea (see Supplementary Table 3, Additional File 1 for the complete list of all reported terms).

**Fig. 3:**
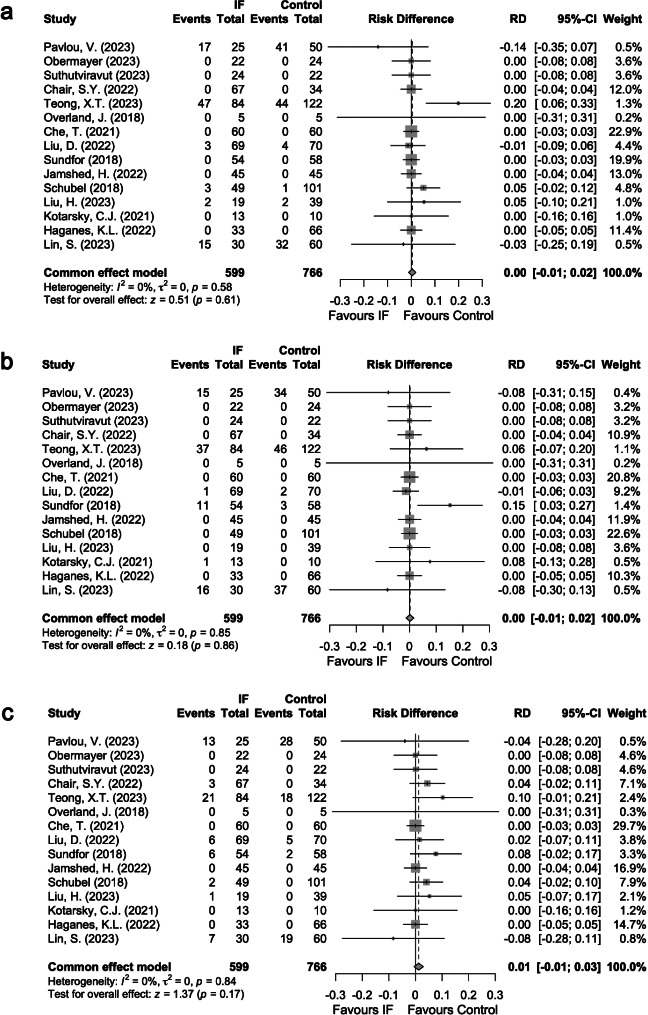
Forest plots in the meta-analysis of IF versus Control in terms of (**a**) fatigue; (**b**) headache; (**c**) dizziness. The sizes of the data markers indicate the relative weight of each study in this analysis. The diamond represents the overall estimated effects in each model. *Note* CI, confidence interval; IF, intermittent fasting; RD, risk difference

Fatigue was reported in 87 (14.5%) subjects in the IF group and 124 (16.2%) subjects in the control group (Supplementary Table 3, Additional File 1). No statistically significant difference in risk between IF and Control was found in the overall pooled analysis (0%, 95%CI: -1% to 2%; *P* = 0.61, Fig. 3a). Similar risk difference in fatigue profile was observed across prespecified subgroups (Supplementary Table 4, Additional File 1).

Headache was reported in 81 (13.5%) subjects in the IF group and 122 (15.9%) subjects in the control group (Supplementary Table 3, Additional File 1). No significant risk difference was equally found in the overall pooled analysis (0%, 95%CI: -1% to 2%; *P* = 0.86, Fig. 3b). The risk of headache profile appeared similar for the two groups when assessed according to subgroups (Supplementary Table 4, Additional File 1).

In addition, 59 (9.8%) subjects in the IF group and 72 (9.4%) subjects in the control group reported dizziness (Supplementary Table 3, Additional File 1). There was no statistically significant difference in the risk between IF and control groups in the overall pooled analysis (1%, 95%CI: -1% to 3%; *P* = 0.17, Fig. 3c). In the IF group versus Control, a numerically greater incidence of dizziness was observed among patients with non-eTREs (2%, 95%CI: -0% to 5%; *P* = 0.07, Supplementary Table 4, Additional File 1) and among patients without diabetes (2%, 95%CI: -0% to 4%; *P* = 0.08, Supplementary Table 4, Additional File 1). The sensitivity analysis after removing the studies by Obermayer et al. and Liu H et al. [25, 33] revealed similar between-group trends in the occurrence rate of dizziness (data not shown).

### Dropouts

**Fig. 4:**
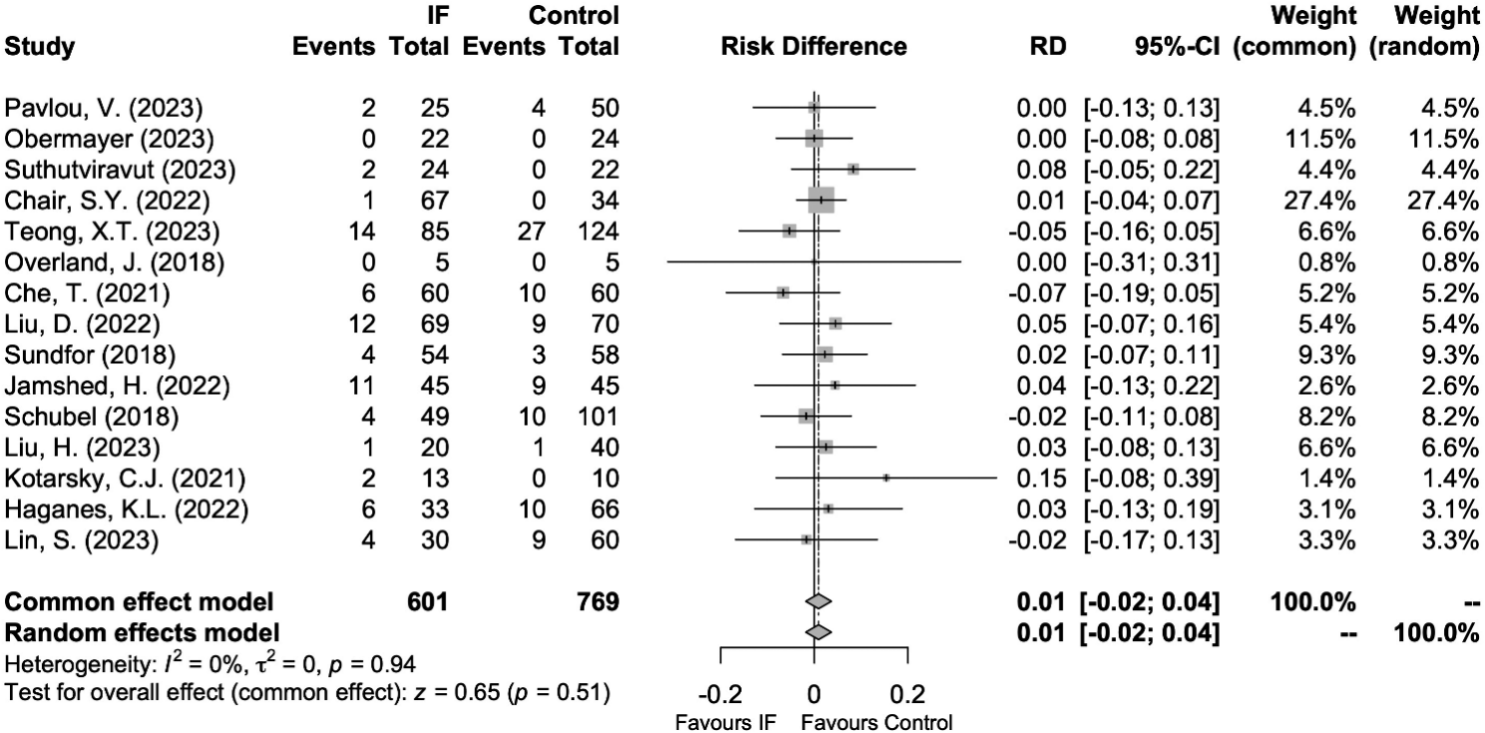
Forest plot in the meta-analysis of IF versus Control on dropout. The sizes of the data markers indicate the relative weight of each study in this analysis. The diamond represents the overall estimated effects in each model. *Note* CI: Confidence Interval; IF: Intermittent Fasting; RD: Risk Difference

All 15 studies reported data on dropout, loss to follow-up, loss due to inability to contact or withdrawal for various reasons. There was no significant difference in the dropout risk between IF and Control (1%, 95%CI: -2% to 4%; *P* = 0.51, Fig. 4). The overall dropout rate was 11.5% in the IF group, which indicated acceptable adherence given the current study treatment duration.

### IF alone versus usual lifestyle

To further understand whether IF alone could increase the occurrence risk of common AEs and dropouts rate compared with the usual diet or standard care (namely neither any diet intervention provided nor food energy intake changed), we extracted a subset of 11 RCTs involving 820 patients meeting the criteria for subgroup analysis. No significant between-group differences were detected in terms of fatigue (1%, 95%CI: -1% to 3%; *P* = 0.55), headache (0%, 95%CI: -2% to 2%; *P* = 0.93) or dizziness (1%, 95%CI: -1% to 4%; *P* = 0.18). It was also noted that in the IF alone group as compared to usual lifestyle, a numerically higher occurrence rate of dizziness was observed among patients with non-eTREs (3%, 95%CI: -0% to 6%; *P* = 0.08, Supplementary Table 5, Additional File 1) and among patients without diabetes (3%, 95%CI: -0% to 6%; *P* = 0.05, Supplementary Table 5, Additional File 1).

### Publication bias

Funnel plots and Egger’s tests revealed no evidence of significant publication bias in the current meta-analysis for fatigue, headache, dizziness or dropout (Egger’s test: *P* = 0.46, 0.11, 0.14 and 0.46, respectively).

### Certainty of the evidence

We assessed the certainty of the evidence of our outcome and found that it was moderate in terms of SAEs or low in the other outcomes, which increased confidence in our effect estimate (Table 2).

**Table 2:** Summary of the certainty of the evidence

No. of studies	Certainty Assessment						No. of subjects		Absolute Effects, RD (95% CI)	Certainty
	Study design	Risk of bias	Inconsistency	Indirectness	Imprecision	Publication bias	Intervention	Control		
Primary outcome: Serious adverse event, binary variable										
15	RCTs	Low	No serious	Serious	No serious		599	766	0.3% vs. 0.5%	⊕⊕⊕
Secondary outcome: Fatigue, binary variable										
15	RCTs	Low	No serious	Serious	No serious	Undetected	599	766	0% (-1%, 2%)	⊕⊕
Secondary outcome: Headache, binary variable										
15	RCTs	Low	No serious	Serious	No serious	Undetected	599	766	0% (-1%, 2%)	⊕⊕
Secondary outcome: Dizziness, binary variable										
15	RCTs	Low	No serious	Serious	No serious	Undetected	599	766	1% (-1%, 3%)	⊕⊕
Secondary outcome: Dropout, binary variable										
15	RCTs	Low	No serious	Serious	No serious	Undetected	599	766	1% (-2%, 4%)	⊕⊕

*Note* CI: Confidence Interval; RCT: Randomized Controlled Trial; RD: Risk Difference. The circles in the certainty column represent the quality of the evidence for each outcome (very low ⊕, low ⊕⊕, moderate ⊕⊕⊕ and high ⊕⊕⊕⊕)

## Discussion

To our knowledge, this is the first meta-analysis to specifically investigate the adverse effects and dropouts of IF compared with the control group in patients with overweight or obesity regardless of diabetes status. Our meta-analysis results suggested that IF was not associated with an increase in the risk of AEs, despite a numerically greater risk of dizziness in the non-eTRE subgroups after treatment with IF. Consistent with our findings on dropouts, another recent meta-analysis [37] revealed no evidence that IF interventions affected dropout in RCTs differently from continuous energy restriction. On the other hand, none of the three recent meta-analyses [9, 10, 37] reported and compared any AEs profile data.

Currently, IF is becoming more popular because it seems to be a simple option to follow in treating several diseases such as overweight and obesity [12]. Like in IF regimens, voluntary abstinence from food has been present throughout human history, such as habits and rituals associated with racial and religious contexts [8]. Hence IF is considered to be safe to some degree, similar to processes with greatly reduced food intake such as hibernation [8]. Very few SAEs (only 2 [0.3%] subjects with IF) were reported in the included RCTs and none of them were judged to be related to the study treatment. None of the 15 involved RCTs reported any major or severe AEs, and 3 of them [25, 26, 35] reported no severe adverse effects. Overall, these data showed that IF regimens are not associated with higher risk of any major AEs when compared to a usual diet or other active comparators.

However, we still found several common AEs (approximately 10% or greater) in the IF group, including fatigue (14.5%), headache (13.5%), constipation (10.2%), dizziness (9.8%) and diarrhea (7.8%). Fatigue is a state of prolonged tiredness, exhaustion, and lack of energy that is not improved by sleep or rest [38]. In general, headache, which is mainly due to hypoglycemia, is a common side effect of fasting [39]. Our data above are apparently in line with the stronger feelings of hunger [30] and desire to eat noted in participants with intermittent energy restriction. In addition, constipation and diarrhea are external gastrointestinal disturbances, which may be caused by irregular TRE regimens compared with habitual eating timing.

Notably, there was a higher greater incidence of dizziness in both subgroups of patients with non-eTRE regimens and without diabetes. Dizziness is primarily caused by a lack of energy and blood volume following fasting and water deprivation [40]. The higher risk of dizziness in the non-eTREs subgroup might originate from a lack of energy intake due to the later eating time, which has been implied in a prior study [41], in which a single case of dizziness was resolved by having a small snack. According to the proposal by Charlot A. et al. [8]. , the food intake should begin at 8 a.m., after the cortisol peak when the activity phase started, and should end no later than 6 p.m., for this purpose of reducing risk of dizziness by obeying the circadian clock. The hypothesized feeding time period started earlier than that in the non-eTRE subgroup, indicating that the non-eTRE may induce a greater risk of dizziness. Therefore, we speculate that early TREs following the circadian clock are beneficial for the prevention of dizziness co-occurring with IF treatment.

Admittedly, our study has several potential limitations. First, it was based on reported aggregate data rather than individual patient data, which may not provide a robust estimation of the comparative risk. The quality of our study relied on the quality of each RCT included. As a result, we only included RCTs in our analysis. RCTs might provide a possibility to estimate the net adverse effect of IF in contrast with the control group when only the usual diet or background treatment shared with the IF group is included in the control group. Second, these 15 RCTs were conducted with different diabetes statuses, various IF regimens, possible concomitant background treatment or physical activity, shorter or longer study treatment durations, multiple countries with diverse dietary cultures, impacts of the recent Coronavirus Disease 2019(COVID-19) pandemic [31, 35] and so on; all of which may represent major potential sources of heterogeneity for our analysis. Therefore, we considered several prespecified subgroup analyses and sensitivity analyses. Nevertheless, we agreed that the certainty of evidence should be low and that the study findings should be applied with caution. Third, none of these 15 RCTs obtained a high score in the Jadad assessment, which was mainly attributed to the unavailability of a double-blinded design due to the nature of the intervention [24–26, 34, 36]. Fourth, grading criteria for AEs was rarely mentioned in included RCTs, let alone severity to be collected and analyzed for AEs. For the purpose of standardized pooling, we performed a medical coding for all AEs terms extracted from included RCTs.

In summary, our meta-analyses showed that IF was not associated with significantly increased risk of AEs in patients with overweight or obesity, regardless of diabetes status, timing and duration of IF regimens. Additional large-scale RCTs stratified by key confounders, matched with circadian clocks and designed to evaluate the long-term effects of various IF regimen were needed to confirm these findings, including AEs profile [8, 42].

Additional file 1: Supplementary Table (1) PRISMA 2020 Checklist. Supplemental Table (2) Key words for literature search on intermittent fasting using PubMed, Embase, Web of Science Core Collection, Cochrane and Clinicaltrials.gov. Supplemental Table (3) Number of studies or subjects with PTs reported in all of 15 included randomized controlled trials. Supplemental Table (4) Subgroup analyses between IF and Control of fatigue, headache and dizziness by pre-defined study characteristics. Supplemental Table (5) Subgroup analyses between IF alone and usual diet of fatigue, headache and dizziness by pre-defined study characteristics. Supplemental Fig. 1. Funnel plots in the meta-analysis of IF versus Control in terms of (a) Fatigue; (b) Headache; (c) Dizziness; (d) Dropout.

### Electronic supplementary material

Below is the link to the electronic supplementary material.

Supplementary Material 1

Supplementary Material 2

Supplementary Material 3

Supplementary Material 4

Supplementary Material 5

## Data Availability

No datasets were generated or analysed during the current study.
