# Supplementary figures and images for: Adverse events profile associated with intermittent fasting in adults with overweight or obesity: a systematic review and meta-analysis of randomized controlled trials
Source: Nutr J. 2024 Jul 10;23:72. doi: 10.1186/s12937-024-00975-9 (PMC11234547; doi:10.1186/s12937-024-00975-9)

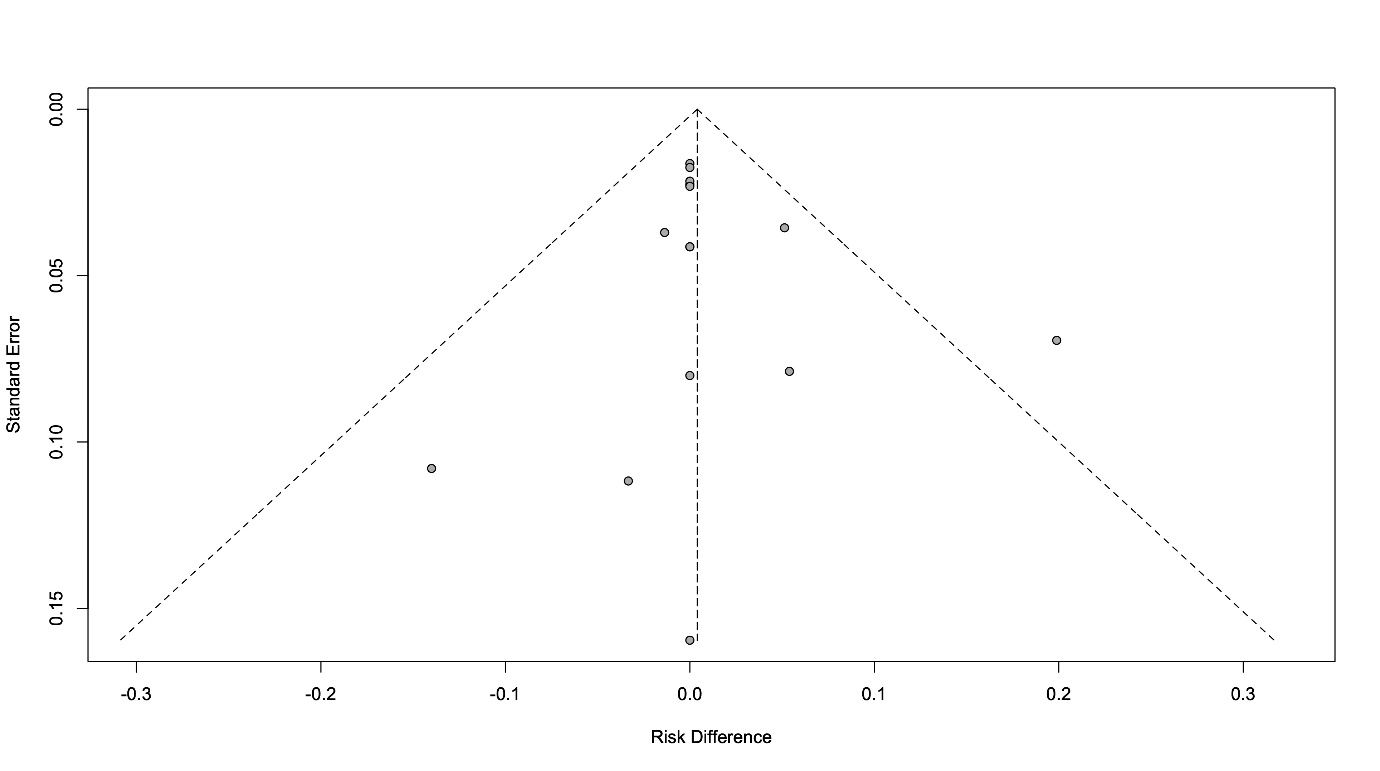


Figure 1a


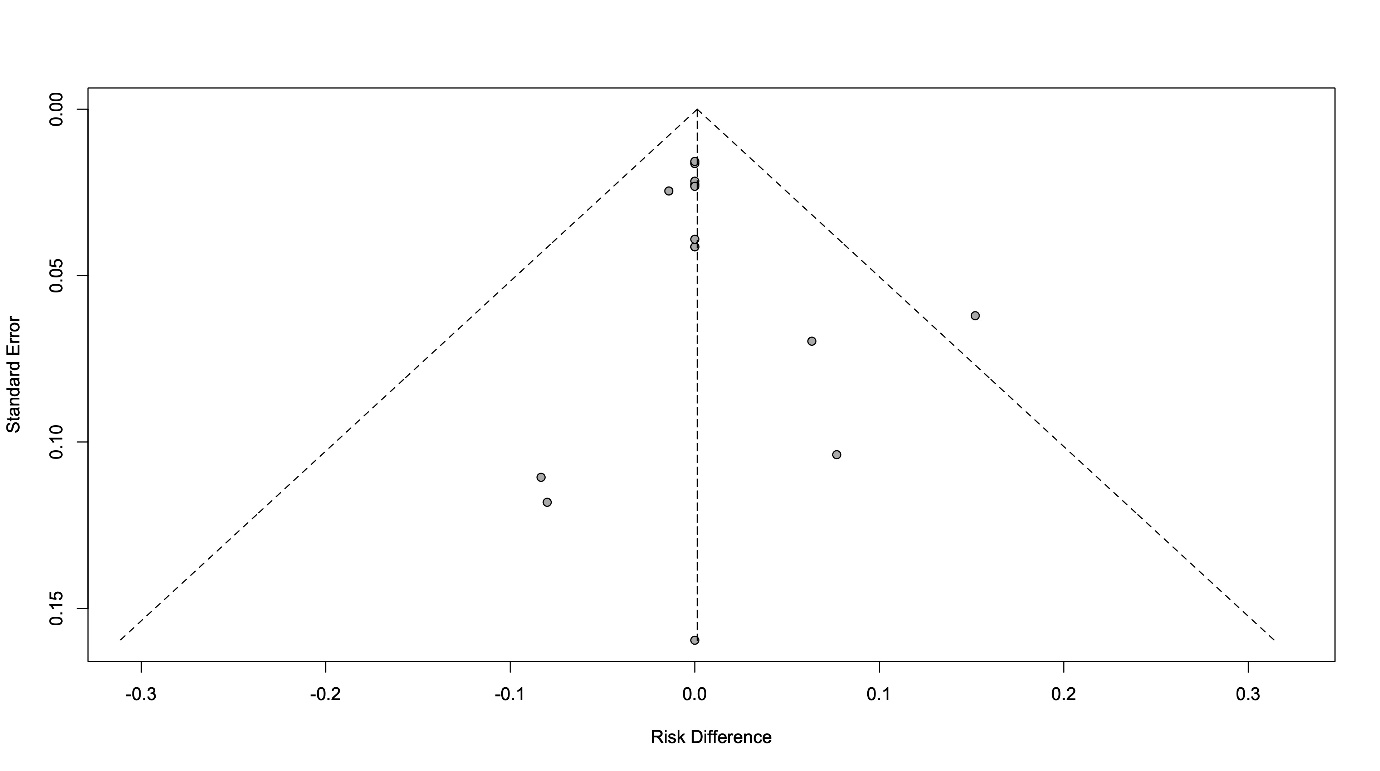


Figure 1b


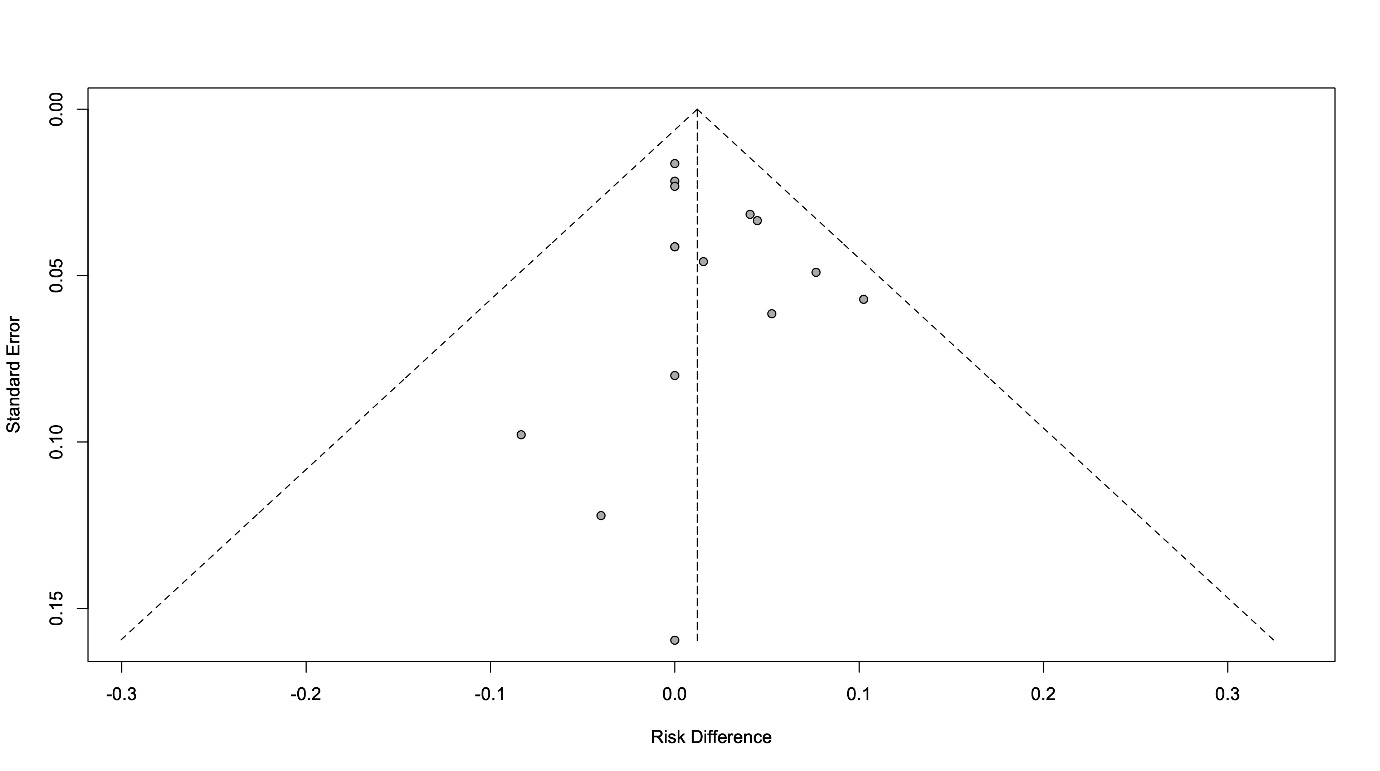


Figure 1c


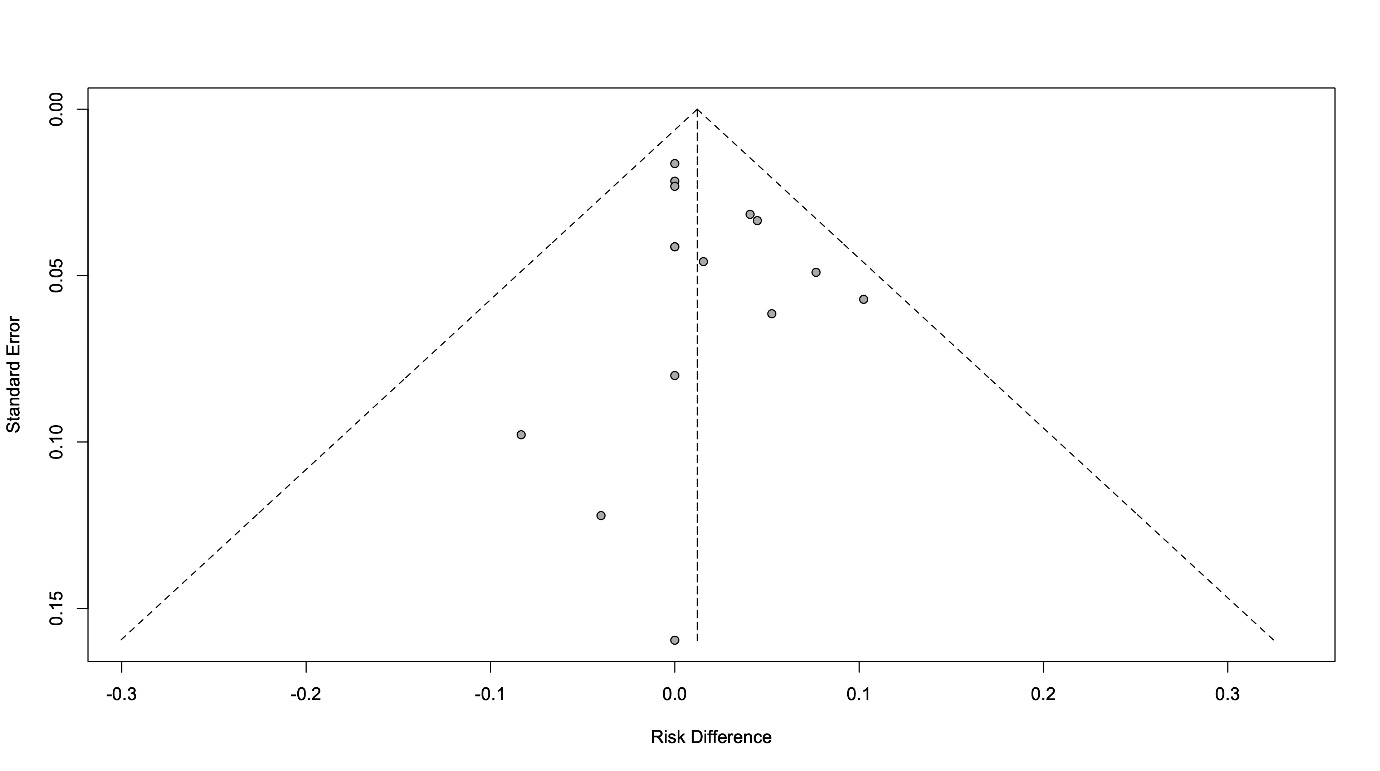


Figure 1d

Supplement: Supplementary file 1 — Supplementary Material 1 [file 12937_2024_975_MOESM1_ESM.docx]
