# Supplementary material for: Adverse events profile associated with intermittent fasting in adults with overweight or obesity: a systematic review and meta-analysis of randomized controlled trials
Source: Nutr J. 2024 Jul 10;23:72. doi: 10.1186/s12937-024-00975-9 (PMC11234547; doi:10.1186/s12937-024-00975-9)
Supplement: Supplementary file 2 — Supplementary Material 2 [file 12937_2024_975_MOESM2_ESM.doc]

**Supplementary Table 2**. Key words for literature search on intermittent fasting using PubMed, Embase, Web of Science Core Collection, Cochrane and Clinicaltrials.gov.

| **Resource** | **Key words for literature search** |
| --- | --- |
| PubMed | (("intermittent fasting"[All Fields] OR "time-restricted feeding"[All Fields] OR "time restricted eating"[All Fields] OR "alternate day fasting"[All Fields] OR "5:2 dieting "[All Fields]) AND (humans[Filter]) AND (randomized controlled trial[Publication Type])) |
| Embase | ('intermittent fasting' OR 'time-restricted feeding' OR 'time-restricted eating' OR 'alternate day fasting' OR '5:2 dieting') AND ('article'/it OR 'article in press'/it OR 'preprint'/it) AND ('randomized controlled trial'/de) |
| Web of Science Core Collection | (TS=("intermittent fasting" OR "time restricted feeding" OR "time restricted eating" OR "alternate day fasting" OR "5:2 dieting")) AND DT==("CLINICAL TRIAL") |
| Cochrane | (intermittent fasting OR time-restricted feeding OR time restricted eating OR alternate day fasting OR 5:2 dieting):kw AND (article):pt |
| Clinicaltrials.gov | intermittent fasting OR time-restricted eating OR time-restricted feeding OR alternate day fasting OR 5:2 dieting | Completed Studies | Studies With Results | Interventional Studies |
