# Supplementary material for: Adverse events profile associated with intermittent fasting in adults with overweight or obesity: a systematic review and meta-analysis of randomized controlled trials
Source: Nutr J. 2024 Jul 10;23:72. doi: 10.1186/s12937-024-00975-9 (PMC11234547; doi:10.1186/s12937-024-00975-9)
Supplement: Supplementary file 3 — Supplementary Material 3 [file 12937_2024_975_MOESM3_ESM.doc]

**Supplementary Table 3. Number of studies or subjects with PTs reported in all of 15 included randomized controlled trials**

| **System organ class**  **Preferred terms** | **Intermittent fasting (N=599)** | | **Control (N=766)** | |
| --- | --- | --- | --- | --- |
|  | **No. of studies reported with PTs** | **No. of subjects with PTs (%)** | **No. of studies reported with PTs** | **No. of subjects with PTs (%)** |
| General disorders and administration site conditions |  |  |  |  |
| **Fatigue** | 6 | 87(14.5%) | 6 | 124(16.2%) |
| Malaise | 1 | 12(2.0%) | 1 | 7(0.9%) |
| Asthenia | 1 | 2(0.3%) | 1 | 1(0.1%) |
| Feeling cold | 1 | 2(0.3%) | 0 | 0 |
| Thirst | 1 | 1(0.2%) | 0 | 0 |
| Nervous system disorders |  |  |  |  |
| **Headache** | 6 | 81(13.5%) | 5 | 122(15.9%) |
| **Dizziness** | 8 | 59(9.8%) | 5 | 72(9.4%) |
| Gastrointestinal disorders |  |  |  |  |
| Constipation | 4 | 61(10.2%) | 4 | 90(11.7%) |
| Diarrhea | 3 | 47(7.8%) | 3 | 78(10.2%) |
| Dry mouth | 2 | 28(4.7%) | 2 | 64(8.4%) |
| Nausea | 3 | 24(4.0%) | 3 | 51(6.7%) |
| Halitosis | 2 | 17(2.8%) | 2 | 38(5.0%) |
| Vomiting | 2 | 13(2.2%) | 2 | 23(3.0%) |
| Dyspepsia | 1 | 5(0.8%) | 1 | 8(1.0%) |
| Upper abdominal pain | 1 | 5(0.8%) | 1 | 4(0.5%) |
| Abdominal pain upper | 1 | 1(0.2%) | 0 | 0 |
| Psychiatric disorders |  |  |  |  |
| Irritability | 3 | 33(5.5%) | 2 | 55(7.2%) |
| Distractibility | 2 | 7(1.2%) | 1 | 1(0.1%) |
| Sleep disorder | 1 | 1(0.2%) | 0 | 0 |
| Metabolism and nutrition disorders |  |  |  |  |
| Hyperglycemia | 1 | 21(3.5%) | 1 | 42(5.5%) |
| Hypoglycaemia | 1 | 10(1.7%) | 2 | 26(3.4%) |
| Decreased appetite | 0 | 0 | 1 | 2(0.3%) |
| Infections and infestations |  |  |  |  |
| Influenza | 1 | 5(0.8%) | 1 | 6(0.8%) |
| Musculoskeletal and connective tissue disorders |  |  |  |  |
| Back pain | 1 | 2(0.3%) | 2 | 5(0.7%) |
| Pain in extremity | 0 | 0 | 1 | 3(0.4%) |
| Vascular disorders |  |  |  |  |
| Peripheral coldness | 1 | 2(0.3%) | 1 | 1(0.1%) |
| Investigations |  |  |  |  |
| Liver function test abnormal | 1 | 1(0.2%) | 0 | 0 |
| Skin and subcutaneous tissue disorders |  |  |  |  |
| Muscle spasms | 1 | 1(0.2%) | 0 | 0 |
| Rash | 0 | 0 | 1 | 1(0.1%) |
| Ear and labyrinth disorders |  |  |  |  |
| Duodenogastric reflux | 0 | 0 | 1 | 1(0.1%) |
| Vertigo | 0 | 0 | 1 | 1(0.1%) |

Adverse events (AEs) reported were coded using Medical Dictionary for Regulatory Activities (MedDRA) 23.1 - English version at PTs and System Organ Class levels. Abbreviations: AEs, adverse events; PTs, preferred terms.
