# Supplementary material for: Adverse events profile associated with intermittent fasting in adults with overweight or obesity: a systematic review and meta-analysis of randomized controlled trials
Source: Nutr J. 2024 Jul 10;23:72. doi: 10.1186/s12937-024-00975-9 (PMC11234547; doi:10.1186/s12937-024-00975-9)
Supplement: Supplementary file 4 — Supplementary Material 4 [file 12937_2024_975_MOESM4_ESM.doc]

**Supplementary Table 4.** Subgroup analyses between IF and Control of fatigue, headache and dizziness by pre-defined study characteristics

| **Study characteristics** | **Number of**  **subjects** | **Fatigue** | | |  | **Headache** | | |  | **Dizziness** | | |
| --- | --- | --- | --- | --- | --- | --- | --- | --- | --- | --- | --- | --- |
|  |  | **RD (95% CI)** | **Test of heterogeneity** | |  | **RD (95% CI)** | **Test of heterogeneity** | |  | **RD (95% CI)** | **Test of heterogeneity** | |
|  |  |  | **I^2^** | **P value** |  |  | **I^2^** | **P value** |  |  | **I^2^** | **P value** |
| Overall | 1,365 | 0%(-1%, 2%) | 0% | 0.58 |  | 0%(-1%, 2%) | 0% | 0.85 |  | 1%(-1%, 3%) | 0% | 0.84 |
| Diabetes mellitus |  |  |  |  |  |  |  |  |  |  |  |  |
| Yes | 251 | 0%(-3%, 3%) | 0% | 0.65 |  | 0%(-3%, 3%) | 0% | 0.93 |  | 0%(-3%, 3%) | 0% | 0.99 |
| No | 1,114 | 1%(-1%, 2%) | 4% | 0.40 |  | 0%(-1%, 2%) | 0% | 0.61 |  | 2%(-0%, 4%) | 0% | 0.66 |
| IF timing |  |  |  |  |  |  |  |  |  |  |  |  |
| Early | 453 | 0%(-2%, 2%) | 0% | 0.96 |  | 0%(-2%, 2%) | 0% | 0.99 |  | 0%(-2%, 3%) | 0% | 0.94 |
| Non-early | 912 | 1%(-1%, 3%) | 22% | 0.24 |  | 1%(-2%, 3%) | 0% | 0.52 |  | 2%(-0%, 5%) | 0% | 0.67 |
| Treatment duration |  |  |  |  |  |  |  |  |  |  |  |  |
| < 6 months | 743 | 0%(-1%, 2%) | 0% | 0.98 |  | 0%(-2%, 2%) | 0% | 1.00 |  | 1%(-1%, 3%) | 0% | 0.95 |
| 6 or 12 months | 622 | 0%(-3%, 3%) | 60% | 0.04 |  | 1%(-3%, 5%) | 50% | 0.09 |  | 4%(-1%, 10%) | 1% | 0.40 |

Abbreviation: CI, confidence interval; IF, intermittent fasting; RD, risk difference.
