# Supplementary material for: Adverse events profile associated with intermittent fasting in adults with overweight or obesity: a systematic review and meta-analysis of randomized controlled trials
Source: Nutr J. 2024 Jul 10;23:72. doi: 10.1186/s12937-024-00975-9 (PMC11234547; doi:10.1186/s12937-024-00975-9)
Supplement: Supplementary file 5 — Supplementary Material 5 [file 12937_2024_975_MOESM5_ESM.doc]

**Supplementary Table 5.** Subgroup analyses between IF alone and usual diet of fatigue, headache and dizziness by pre-defined study characteristics

| **Study characteristics** | **Number of**  **subjects** | **Fatigue** | | |  | **Headache** | | |  | **Dizziness** | | |
| --- | --- | --- | --- | --- | --- | --- | --- | --- | --- | --- | --- | --- |
|  |  | **RD (95% CI)** | **Test of heterogeneity** | |  | **RD (95% CI)** | **Test of heterogeneity** | |  | **RD (95% CI)** | **Test of heterogeneity** | |
|  |  |  | **I^2^** | **P value** |  |  | **I^2^** | **P value** |  |  | **I^2^** | **P value** |
| Overall | 820 | 1%(-1%,3%) | 0% | 0.50 |  | 0%(-2%,2%) | 0% | 0.99 |  | 1%(-1%,4%) | 7% | 0.37 |
| Diabetes mellitus |  |  |  |  |  |  |  |  |  |  |  |  |
| Yes | 241 | -0%(-3%, 3%) | 0% | 0.63 |  | -0%(-3%, 3%) | 0% | 0.96 |  | 0%(-3%, 3%) | 0% | 0.95 |
| No | 579 | 1%(-1%, 4%) | 0% | 0.34 |  | 0%(-2%, 2%) | 0% | 0.95 |  | 3%(-0%, 6%) | 20% | 0.67 |
| IF timing |  |  |  |  |  |  |  |  |  |  |  |  |
| Early | 224 | 0%(-3%, 3%) | 0% | 0.80 |  | 0%(-3%, 3%) | 0% | 1.00 |  | 0%(-3%, 3%) | 0% | 0.71 |
| Non-early | 596 | 1%(-2%, 4%) | 20% | 0.27 |  | 0%(-2%, 2%) | 0% | 0.95 |  | 3%(-0%, 6%) | 21% | 0.26 |
| Treatment duration |  |  |  |  |  |  |  |  |  |  |  |  |
| < 6 months | 561 | 1%(-1%, 3%) | 0% | 0.91 |  | 0%(-2%, 2%) | 0% | 1.00 |  | 1%(-1%, 3%) | 0% | 0.88 |
| 6 or 12 months | 259 | 4%(-8%, 17%) | 68% | 0.04 |  | 1%(-12%, 14%) | 0% | 0.44 |  | 9%(-1%, 18%) | 65% | 0.06 |

Abbreviation: CI, confidence interval; IF, intermittent fasting; RD, risk difference.
